# Addressing Suicide Risk in Patients Living With Dementia During the COVID-19 Pandemic and Beyond

**DOI:** 10.1093/geront/gnac042

**Published:** 2022-04-02

**Authors:** Elena Portacolone, Amy L Byers, Jodi Halpern, Deborah E Barnes

**Affiliations:** Institute for Health & Aging, University of California San Francisco, San Francisco, California, USA; Philip Lee Institute for Health Policy Studies, University of California San Francisco, San Francisco, California, USA; School of Medicine, University of California San Francisco, San Francisco, California, USA; San Francisco Veterans Affairs Health Care System, San Francisco, California, USA; University of California Berkeley-University of California San Francisco Joint Medical Program, School of Public Health, University of California, Berkeley, California, USA; School of Medicine, University of California San Francisco, San Francisco, California, USA

**Keywords:** Health care policy, Mental health, Physician–patient communication/relationships, Quality of care, Technology

## Abstract

Alzheimer’s disease and related dementias (ADRD) are progressive illnesses characterized by decline in cognitive function that impairs performing daily activities. People with ADRD are at an increased risk of suicide, especially those who have comorbid mental health conditions, have specific types of ADRD, or have been recently diagnosed. The coronavirus disease 2019 (COVID-19) pandemic has increased the distress of people with ADRD, a population also at increased risk of contracting the COVID-19 virus. In this article, we draw on a case study and use the Interpersonal Theory of Suicide to help describe the association between ADRD and suicide risk. Secondly, we call for new strategies to mitigate suicide risk in people living with ADRD during and beyond the current pandemic by using lessons learned from cancer care. Our goal is not to dictate solutions but rather to start the conversation by outlining a framework for future research aimed at preventing death by suicide in people with ADRD. Specifically, we draw on the updated Framework for Developing and Evaluating Complex Interventions to reflect on the complexity of the issue and to break it down into achievable parts to reduce the risk of suicidal behavior (ideation, plans, attempts) in those living with ADRD.

“If it turns out that there is no quality to my life … I’m not going to be around for that. I just really am not. I would like to have some way of just saying, well, that’s it. Wrap it up.” Less than 1 year after saying these words, Ms. Reds (a pseudonym), a 72-year-old widow whose memory was so impaired that she could not recall her home address, died of suicide. A former manager with a large circle of friends and two adult children, she followed the instructions in a book, placing her head in a helium balloon, and died. Neither her obituary nor her memorial service mentioned her suicide. Her case came to light because she was participating in our research on living alone with Alzheimer’s disease and related dementias (ADRD; Portacolone et al., 2019a, 2019b). During four interviews in her bright living room, Ms. Reds revealed her feelings of despair in experiencing the symptoms of ADRD. Her distress was compounded by interactions with her primary care physician and her neurologist, whom she felt provided little explanation or empathy for her condition. The last visit with the neurologist disappointed her because he spoke only with her adult child. Ms. Reds noted, “No one said much to me … I mean, nobody asked me anything.”

Ms. Reds’ suicide, unfortunately, is more common than we would hope. Although studies are mixed about ADRD being a risk factor for death by suicide, and some studies have even found a reduced overall risk of suicide in patients with ADRD (Erlangsen et al., 2020) there is clear evidence of subgroups of patients with ADRD whose risk of suicide is elevated. These include patients who are younger, depressed, or are newly diagnosed in the early stages of ADRD or with mild cognitive impairment (Diehl-Schmid et al., 2017; Gunak et al., 2021; Serafini et al., 2016).

The coronavirus disease 2019 (COVID-19) pandemic has further contributed to social isolation and psychological distress in older adults with ADRD (Burns et al., 2021; Talbot & Briggs, 2021), a population particularly at risk to contract the COVID-19 virus (Liu et al., 2020; Wang et al., 2021). Reported distress has included a sense of abandonment, fear, and isolation (Barguilla et al., 2020; Burns et al., 2021; Giebel et al., 2022; Talbot & Briggs, 2021). Through our own study on adults with ADRD living alone (Portacolone et al., 2021), we have seen how sheltering in place during COVID-19 caused extreme isolation and psychological distress. Furthermore, psychological distress of older adults with ADRD stems from the absence of treatments for ADRD and fear of becoming overly dependent on others (Cipriani et al., 2013; Conejero et al., 2018).

In this article, our goal is to take the first step in starting the conversation about how to improve care and reduce suicide risk. To do this, we first discuss the potential connection between symptoms of ADRD and suicide using the Interpersonal Theory of Suicide (Van Orden et al., 2010). We then discuss empirical data for the association between ADRD and suicide, and relate deficits in the detection of and mitigation of suicide risk with ADRD to gaps in how cancer was managed several decades ago, providing four lessons learned from the evolution of cancer care. Finally, we draw on the updated Framework for Developing and Evaluating Complex Interventions (Skivington et al., 2021b) to outline research priorities and next steps to address risk of suicidal behavior (ideation, plans, attempts) and death by suicide among people with ADRD during the COVID-19 pandemic and beyond.

## Interpersonal Theory of Suicide and ADRD

Suicide is often a response to a need to relieve *unbearable mental pain* characterized by a sense of alarm, brokenness, and disconnection, as well as anguish, sorrow, loss, and dread (Mann, 1998; Orbach, 2011). An important trigger can be observing negative changes in one’s self, with a sense of irreversibility and loss of control. Feelings of defeat and hopelessness with regard to specific problems often accompany suicidal behavior (Orbach, 2011).

The symptoms of ADRD—memory loss, personality changes, disorientation, trouble with thinking, impaired judgment—can threaten core identity and may lead to suicidal behavior (Cantor, 2018). In the absence of treatment that can reverse ADRD, the inevitability of worsening symptoms can spark despair. When people find themselves having difficulty finding even common words or getting lost in familiar places, they can become frightened about their future capacity. They may be afraid of becoming overly dependent upon others. They may also begin avoiding others to hide their symptoms, or they may be shunned by others who do not understand or are afraid of their symptoms, leading to social isolation. Whereas most people contemplate suicide to escape a present reality, those with ADRD may be more likely to use suicide as an escape to avoid a *projected* reality where they imagine themselves stripped of dignity and independence.

According to the Interpersonal Theory of Suicide (Van Orden et al., 2010), social connectedness has a critical role in preventing death by suicide. Specifically, this theory suggests that “the most dangerous form of suicidal desire” (2010, p. 2) is caused by the simultaneous presence of two constructs that are interpersonal, thus influenced by interactions with others. One is perceived burdensomeness, which is a mental state characterized by the “belief that the self is so flawed as to be a liability to others,” with a related belief that others would “be better off if I were gone” (Van Orden et al., 2012, p. 855). The second interpersonal construct is a thwarted sense of belonging which manifests when the fundamental human psychological need to belong is unmet (Baumeister & Leary, 1995).

The Interpersonal Theory of Suicide further separates the *desire* to engage in suicidal behavior from the *capability* to engage in such behavior, with most people who have the desire to die by suicide not possessing the capability to act upon their desire. However, when someone has both desire and capability, then death by suicide is likely. For example, according to Ms. Reds’ son, on numerous occasions his mother announced to her adult children her intention to die by suicide with the argument that she did not want to become as incapacitated as her father. Ms. Reds also meticulously planned her own memorial, from the order of the speeches to the choice of food. Additionally, her son explained that his mother gained the expertise to die by suicide by studying the methods in a specialized book and she sought a friend’s help to follow the instructions, while making sure that the friend would not be incriminated after her passing. To this point, one of the first assertions of Ms. Reds during our study was: “I’ve always been a person who organizes things.”

## ADRD, Suicide, and Four Applicable Lessons Learned From the Evolution of Cancer Care

Several recent systematic reviews have found that specific subgroups of people with cognitive impairment or ADRD are more likely to die by or to consider suicide (Diehl-Schmid et al., 2017; Draper, 2015; Erlangsen et al., 2020; Serafini et al., 2016). Specifically, patients who are younger or have psychiatric comorbidities, particularly depression, and who have attempted suicide previously, have an increased suicide risk (Annor et al., 2019; Diehl-Schmid et al., 2017; Draper, 2015; Lara et al., 2015; Sabodash et al., 2013; Serafini et al., 2016). Semantic dementia (Diehl-Schmid et al., 2017; Sabodash et al., 2013) and behavioral variant frontotemporal dementia (bvFTD; Zucca et al., 2019) have been identified as potentially creating greater risk than other forms of dementia. Primary symptoms of semantic dementia include inability of retrieving words and remembering the meaning of specific words, which are often accompanied by emotional withdrawal, depression, apathy, or irritability (Hodges & Patterson, 2007). Emerging evidence suggests that in patients with semantic dementia, language impairments exacerbate psychiatric comorbidities, which, in turn, aggravate language impairments, which is a “vicious circle” likely increasing death by suicide (Costanza et al., 2021). With regard to bvFTD, primary symptoms include impulsivity and social disinhibition often accompanied by irritability and lack of empathy (Pressman & Miller, 2014). However, why people with bvFTD and are more prone to death by suicide is not well characterized (Zucca et al., 2019). Furthermore, the risk of suicide in patients with ADRD is greatest shortly after diagnosis (Annor et al., 2019; Diehl-Schmid et al., 2017; Draper, 2015; Erlangsen et al., 2020; Gunak et al., 2021).

Receiving a diagnosis of ADRD is often a profound, life-changing event, with many similarities to receiving a diagnosis of cancer. Both disorders have been associated with heightened risk of suicide largely related to a recent diagnosis (Ahn et al., 2015; Cipriani et al., 2013; Conejero et al., 2018; Henson et al., 2018). We propose that deficits in the detection of suicide risk factors and in follow-up systems of care for patients with ADRD today are similar to how cancer was managed several decades ago. Thus, we can take the emphasis on people’s projection that they will be a burden, and fear of isolation so central to ADRD, and lessons learned from the evolution of cancer care, where similar fears arose, to suggest *four lessons learned to improve care for patients with ADRD*.

First, the evasive style of communication of medical providers regarding an ADRD diagnosis today is in some ways reminiscent of communication regarding cancer in the 60s and 70s. In those times, the “C” word was rarely heard in medical encounters because of limited treatments, as well as stigma (Gordon, 1997). Owing to progress in cancer treatments and campaigns against stigma, medical providers now openly discuss cancer treatments with patients and their families (Rowland et al., 2013), thus strengthening patient/provider relationships and fostering social connections, supporting a sense of belonging.

Similar to past cancer practices, providers today are often reluctant to discuss the “D” word (Chong et al., 2016; Swallow, 2017), because they do not want to upset patients and their families about a condition that lacks effective treatments. According to research by our group (Portacolone et al., 2018, 2019b), this vacuum of information likely facilitates dread and isolation, especially for those who notice symptoms of cognitive impairment and have many unanswered questions. For example, Ms. Reds openly expressed her frustration with a lack of knowledge of the gravity of her condition. In her words:

I would like to have an assessment by somebody at the clinic that would say, on the continuum of people that are having memory problems or Alzheimer’s or whatnot—I don’t know when one thing turns to the other thing—I’d like to have somebody give me some information so that it would be clearer to me exactly where am I at on this continuum on this process of losing my mind?

She participated in clinical trials on dementia to help science move forward as well as to get accurate information about her status, which she never received.

Second, the limited treatment options for patients with ADRD today evoke the dim horizons of cancer patients several decades ago. Although cancer used to nearly always be a fatal diagnosis, today many cancer patients have a variety of treatment options available that, in many cases, may result in long-term remission (Jacobs et al., 2009; Rosenberg & Partridge, 2017). In contrast, patients with ADRD have few treatment options available and, with the exception of some rare reversible causes of ADRD, no potential for remission or reversal of symptoms, which may exacerbate feelings of despair. What most separates ADRD from cancer, and heightens the risk of suicide, is that patients with ADRD may feel great urgency to take action earlier in the disease process while they still maintain the cognitive capacity to do so. Ms. Reds’ son summarized this dynamic for his mother, “If she had waited too long to take her life, then she wouldn’t have [had] the capacity to do that.”

Third, the isolation of patients with ADRD diagnoses today recalls the isolation surrounding patients with cancer decades ago. Fortunately, cancer care has evolved over the past several decades, and today there is now often an explicit team-based effort to create support around newly diagnosed persons as the slogan “No-one should face cancer alone” attests (Macmillan Cancer Support, 2014; Mankell, 2014). This team support fosters a sense of social cohesion and belonging. In contrast, people with ADRD are typically diagnosed relatively late in the disease process and are often quite isolated as they try to cope with their symptoms (Holwerda et al., 2014; Portacolone et al., 2018, 2019b; Spreadbury & Kipps, 2019). The stigma attached to ADRD further isolates them (Lopez et al., 2019; Portacolone, 2018; Spreadbury & Kipps, 2019), and this isolation has been further exacerbated by the new restrictions brought forth by the COVID-19 pandemic. According to the authors of the Interpersonal Theory of Suicide, social isolation is “arguably” one of “the strongest and most reliable predictor(s) of suicidal ideation, attempts, and lethal suicidal behavior” (Van Orden et al., 2010, p. 5).

Fourth, both ADRD and cancer have strong associations with depression and with suicidal ideation as a symptom of major depression (Draper, 2015; Lara et al., 2015; Seyfried et al., 2011). However, whereas patients diagnosed with depression and cancer usually have full access to mental health services (Badr, 2016), a diagnosis of ADRD can jeopardize access to these essential services in depressed patients in some health care settings (Dick-Muehlke, 2016). In the United States, access might be negated because of erroneous interpretations of reimbursement policies in the public health insurance programs for older adults (Medicare) and low-income individuals (Medicaid). For example, in California, Medicaid-eligible patients qualify for specialty mental health services if they “*will improve* as a result of treating the condition [e.g., depression], which would not be responsive to physical health care” (Dick-Muehlke, 2016). Because providers often incorrectly assume that the depression of patients with ADRD will not improve, essential services are overlooked (Dick-Muehlke, 2016). For example, in our longitudinal study on living alone with ADRD, the two participants who expressed suicidal ideation prior to the COVID-19 pandemic received no specialized mental health support in the midst of the pandemic (Portacolone et al., 2021). Instead, one was given (by her social worker) “toy blocks to keep my hands busy and to stop me from thinking too much.”

There is emerging evidence that patients with ADRD can engage in, and often benefit from psychotherapy, including cognitive behavioral, stimulation, and psychodynamic therapies, as well as pharmacotherapy and relaxation training (Johnston & Narayanasamy, 2016; Orgeta et al., 2014; Reuben et al., 2019; Sorensen et al., 2006; Woods et al., 2006). Unfortunately, mental health providers and neurologists are seldom trained to provide supportive services to persons with ADRD (Carpenter & Dave, 2004). As a result, depression and other mental health conditions in persons with ADRD are often left untreated, hampering an opportunity for them to come to terms with their chronic condition, which may contribute to their suicide risk (Annor et al., 2019; Seyfried et al., 2011).

### Reflections for Moving Forward

**Figure 1.**
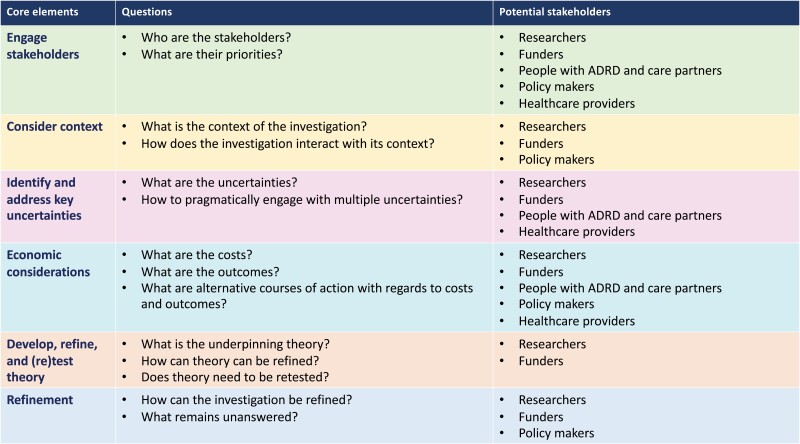
The Core Elements of the Skivington et al. Research Framework. *Note*: ADRD = Alzheimer’s disease and related dementias.

Addressing thoughts of suicide and preventing death by suicide in people with ADRD is a complex endeavor requiring research at multiple levels, ranging from intervention work to policy development. To start to reflect about the complexity of the issue and how to break it down into achievable parts to reduce the risk of suicide in those diagnosed with ADRD, we draw on the updated Framework for Developing and Evaluating Complex Interventions (Skivington et al., 2021b) developed by the United Kingdom’s Medical Research Council. The original framework was published in 2000 and has been widely used to inform development of complex interventions (Skivington et al., 2021a). The 2021 update incorporates conceptual, methodological, and theoretical learnings and is designed to help researchers develop and evaluate complex interventions with input from diverse stakeholders and using appropriate methods. We draw from this framework because its reflective and broad perspective aims at maximizing the efficiency and impact of research. Specifically, the framework invites researchers to consider “core elements” and raise questions about the context, costs, and uncertainties of specific investigations, as well as the stakeholders involved, and the overall impact (Figure 1). Even though Skivington et al. developed this framework to guide complex interventions, its core questions are relevant as a guide for all types of investigations.

Using this framework as a guide, we propose that the next step in preventing death by suicide in people with ADRD is to conduct *reflective* research that engages stakeholders (e.g., people with ADRD, care partners, policy makers, funders, health care providers, researchers), identifies uncertainties, takes economic burden into consideration, tests and refines theory, and has a far-reaching impact. This could begin by convening roundtables and meetings that include diverse perspectives from key stakeholders. Researchers could be further engaged by organizing symposia or featured research sessions at scientific meetings of geriatric, ADRD, mental health and suicide prevention professional organizations. For example, drawing lessons from cancer care, in October 2021 Skivington was invited to discuss his theory at a research symposium on cancer (University of Bristol, 2021). To begin the conversation, we provide our initial reflections on how some of the core elements of the framework might apply to investigations to reduce death by suicide in people living with ADRD.

#### Engage stakeholders

Right now, key stakeholders include researchers, policy makers, and officers of prominent research institutions such as National Institutes of Health whose priorities include advancing science to enhance health and lengthen lives (2022). It will be essential to include people with ADRD, caregivers, and health care providers, comprising primary care and mental health providers.

#### Consider context

Context includes multiple facets, beginning with the psychological concerns of people with ADRD, as well as their characteristics, such as race/ethnicity, socioeconomic status, health insurance, immigration status, sexual orientation, living arrangements, comorbidities, and access to lethal means. Context also includes the health care settings, systems, regulations, policies, and barriers to accessing essential services (e.g., mental health, telehealth, home care services), as well as prevailing ideologies, such as the relevance of independence in western societies. Finally, context includes the ongoing COVID-19 pandemic that has exacerbated social isolation and distress in people with ADRD, as well as accelerated progress in the provision of services via telehealth (Gray et al., 2020; Torous & Wykes, 2020). A promising avenue of further investigations stems from evidence of decreased anxiety and depression among patients with ADRD visited by providers using telehealth during the pandemic (Weiss et al., 2021).

#### Identify and address key uncertainties

Major uncertainty is whether and how death by suicide occurs. From the perspective of the person with ADRD planning death by suicide, an uncertainty is when to act before the ADRD has progressed to the point that one is unable to complete the plan. From the providers’ perspective, it is uncertain *when* is the best time to screen for thoughts of suicide, *how* to screen, and *who* should be screened: all people with ADRD or only those more at risk? Also, the specific parameters of the screenings are not defined. Yet another major uncertainty stems from the limited knowledge on what therapies are effective to prevent death by suicide in general, let alone among people with ADRD (Fox et al., 2020). Current treatments focus on addressing depression, and providing mental health services, without a specific emphasis in suicide prevention. For example, emerging evidence from cancer care points to the role of the “psycho-oncologist” in reducing distress and depression (not death by suicide) among patients with cancer of the bladder (Bultz & Walker, 2020). This evidence builds from a large body of research in psycho-oncology (Fawzy, 1999; Holland, 2001, 2018). With regard to patients with ADRD, it is unclear whether their deaths by suicide are facilitated by limited availability of mental health services, limited appropriateness of existing mental health services, and/or something else. Furthermore, there is a limited understanding on barriers to providing treatments in terms of policies and training focused on specific characteristics of ADRD patients. For example, Ms. Reds talked with a psychologist who did not address her distress appropriately, as her recollections below illustrate. Her words were audio-recorded 5 months before her carefully planned death:

He [the psychologist] says to me, “You’ve got to have a different string on your violin. You’ve been playing this same one that says, ‘I’m losing my memory, I’m losing my memory, I’m losing my memory, I’m losing my memory. Stop it! That is not helpful. Something more could be so much more productive. So work on the other. Find these other things that are going on that are perfectly great and useful and helpful and are positive.” … And I told him, “You know, that is going to be so difficult for me. I don’t know how to go about doing that. Because I have spent years with somebody who was losing their memory. It’s a fact that I am losing my memory.”

#### Economic considerations

Screenings, targeted intervention and treatments, as well as specific public policies to expand access to services to people with ADRD (e.g., home care aides trained in suicide prevention) require consistent financial and human resources. To understand the best allocation of resources, the framework invites researchers to conduct comparative analyses of alternative courses of actions with consideration to costs and outcomes. The early involvement of economists in research would support the focus on economic considerations. Furthermore, Skivington et al. invite the use of broad ranging approaches such as cost–benefit or consequence analysis because they capture a full range of costs and benefits.

#### Develop, refine, and (re)test theory

We have suggested an initial theory to put to the test—the Interpersonal Theory of Suicide. To prevent death by suicide in people with ADRD, we propose that future research should understand how to identify people with ADRD who possess both desire and capability to engage in suicidal behaviors such as Ms. Reds. Relatedly, in order for providers to identify patients with ADRD at risk, it is important to measure desire and capability to die by suicide, as well as perceived burdensomeness and thwarted sense of belonging. In order to better understand the role of ADRD in increasing the vulnerability to death by suicide, it is also important to identify variations of these constructs in those with ADRD. Furthermore, to refine our understanding of how these constructs are expressed differently for those with ADRD, we propose comparisons between patients with and without ADRD, as well as between patients with ADRD versus those with cancers that currently lack effective treatments.

Taken all together, these are only a few examples of the constellation of questions and directions elicited by a reflective use of the Skivington et al. framework. Sparking new investigations is critical to reduce psychological distress and prevent the tragedy of suicide in patients who, like Ms. Reds, might still have many years of good quality of life.
